# Detection of Hippocampal Subfield Asymmetry at 7T With Automated Segmentation in Epilepsy Patients With Normal Clinical Strength MRIs

**DOI:** 10.3389/fneur.2021.682615

**Published:** 2021-11-15

**Authors:** Akila Pai, Lara V. Marcuse, Judy Alper, Bradley N. Delman, John W. Rutland, Rebecca E. Feldman, Patrick R. Hof, Madeline Fields, James Young, Priti Balchandani

**Affiliations:** ^1^Icahn School of Medicine at Mount Sinai, New York, NY, United States; ^2^Department of Neurology, Icahn School of Medicine at Mount Sinai, New York, NY, United States; ^3^BioMedical Engineering and Imaging Institute, Icahn School of Medicine at Mount Sinai, New York, NY, United States; ^4^Department of Radiology, Icahn School of Medicine at Mount Sinai, New York, NY, United States; ^5^Translational and Molecular Imaging Institute, Icahn School of Medicine at Mount Sinai, New York, NY, United States; ^6^Department of Computer Science, Math, Physics, and Statistics, University of British Columbia, Okanagan, BC, Canada; ^7^Nash Family Department of Neuroscience and Friedman Brain Institute, Icahn School of Medicine at Mount Sinai, New York, NY, United States

**Keywords:** 7T MRI, epilepsy, hippocampus, subfield, volumetry, asymmetry

## Abstract

While the etiology of hippocampal sclerosis (HS) in epilepsy patients remains unknown, distinct phenotypes of hippocampal subfield atrophy have been associated with different clinical presentations and surgical outcomes. The advent of novel techniques including ultra-high field 7T magnetic resonance imaging (MRI) and automated subfield volumetry have further enabled detection of hippocampal pathology in patients with epilepsy, however, studies combining both 7T MRI and automated segmentation in epilepsy patients with normal-appearing clinical MRI are limited. In this study, we present a novel application of the automated segmentation of hippocampal subfields (ASHS) software to determine subfield volumes of the CA1, CA2/3, CA4/DG, and the subiculum using ultra high-field 7T MRI scans, including T1-weighted MP2RAGE and T2-TSE sequences, in 27 patients with either mesial temporal lobe epilepsy (mTLE) or neocortical epilepsy (NE) compared to age and gender matched healthy controls. We found that 7T improved visualization of structural abnormalities not otherwise seen on clinical strength MRIs in patients with unilateral mTLE. Additionally, our automated segmentation algorithm was able to detect structural differences in volume and asymmetry across hippocampal subfields in unilateral mTLE patients compared to controls. Specifically, amongst unilateral mTLE patients with longer disease durations, volume loss was observed in the ipsilateral CA1 and CA2/3 subfields and contralateral CA1. There were no differences in subfield volumes in patients with NE compared to controls. We report the first application of 7T with automated segmentation to characterize the relationship between disease duration burden and asymmetry across specific hippocampal subfields in this population. Disease duration was found to have a statistically significant positive relationship with subfield asymmetry within the unilateral mTLE cohort. These findings highlight the ability of 7T MRI and automated segmentation to provide novel qualitative and quantitative information in epilepsy patients who are otherwise MRI-negative at clinical field strengths.

## Introduction

Epilepsy is a common neurological disorder characterized by recurrent, unprovoked seizures. While first-line therapy for adult focal epilepsy includes anti-seizure medication (ASMs), up to 30% of patients are refractory to ASM or develop ASM resistance (1) and may be eligible for neurosurgical intervention (2). The utilization of magnetic resonance imaging (MRI) with clinical and electrophysiologic data in pre-surgical planning, has increased rates of successful seizure foci resection due to *in vivo* identification of hippocampal sclerosis (HS) as defined by atrophy and increased signal intensity within the hippocampus on T2-weighted images (3–7). Specifically, histopathological studies have confirmed that patients with specific phenotypes of HS, which vary based on involvement of different hippocampal subfields, are more likely to have better seizure-free outcomes than others (4, 8). However, up to 20–30% of patients with refractory focal epilepsy are “MRI-negative,” or do not have identifiable HS or focal lesions, at conventional 1.5 or 3 Tesla (T) MRI field strengths (9, 10) and are less likely to be seizure free (11). Developing methods to identify hippocampal abnormalities and the distribution of subfield neuronal cell loss in “MRI-negative” patients may provide valuable information including the expected clinical course and associated surgical outcomes.

Considerable effort has been directed toward developing hippocampal subfield segmentation techniques in order to elucidate subtle volumetric changes that may not otherwise be detected by visual inspection at clinical field strengths. Multiple studies have validated automated segmentation techniques in patients that are “MRI-positive” at clinical field strengths and has been shown to identify and quantify hippocampal subfield atrophy (12–14). In patients with lateralized mesial temporal lobe epilepsy (mTLE), automated segmentation has identified specific patterns of HS in MRI-positive patients including significant volume reductions in subfields ipsilateral to the suspected seizure onset zone (sSOZ) such as the CA1, CA2/3, CA4/DG, and subiculum (12–14). However, in “MRI-negative” patients, automated volumetric segmentation methods alone have not had sufficient sensitivity to identify patterns of hippocampal subfield volume reduction (14). Since volumetric segmentation has not been shown to independently identify patterns of hippocampal atrophy at conventional field strengths, recent studies have attempted to utilize higher resolution imaging to identify patterns across hippocampal subfields (15–18).

In October 2017, the 7 Tesla (7T) Terra MRI scanner (Siemens Healthcare, Erlangen, Germany) received 510 K approval from the Food and Drug Administration and is becoming increasingly available as a clinical tool. 7 Tesla MRI scanners offer increased resolution and contrast enabling more accurate and reliable delineation of intricate structures including hippocampal subfields (17, 19). Preliminary studies characterizing volumetric atrophy or asymmetry with 7T in “MRI-negative” patients, or patients with normal appearing MRIs at clinical field strengths, are limited. Imaging at 7T combined with manual segmentation techniques has not detected trends in volumetric atrophy consistent with well-established patterns of HS (16, 17). Recently, in a study of nine MRI-negative patients mTLE, the combined application of 7T with automated segmentation showed a very slight whole hippocampus asymmetry which was not significant at the level of hippocampal subfields (18). Imaging at 7T combined with automated segmentation has not yet been investigated in larger cohorts, in bilateral disease, and epilepsy patients with neocortical onsets.

In this paper, we present the application of a novel technique including 7T MRI combined with automated hippocampal subfield segmentation to characterize hippocampal subfield volumes and asymmetry patterns and their relationship to disease duration in epilepsy patients and age-matched, gender-matched controls. We hypothesize that this combined approach may help elucidate patterns in hippocampal subfield atrophy and asymmetry in patients who were previously MRI-negative at conventional field strengths.

## Materials and Methods

### Participants

After this study was approved by the Institutional Review Board at Mount Sinai Hospital, we recruited 27 patients (mean age of 31 years, 13 females, 14 males) with either mTLE (*n* = 16) or neocortical epilepsy (NE) (*n* = 11). Of note, four of the patients with mTLE also had ipsilateral neocortical involvement (mTLE+). As these patients had mesial temporal onsets potentially affecting their subfield volumes, they were included in the mTLE group. Nine of the patients had bilateral onsets (three mTLE and six NE). Patients were age and gender matched (±4 years) with healthy controls (mean age of 31.7 years, 13 females, 14 males). Written informed consent was obtained from all participants at the beginning of the study. All patients had non-lesional diagnostic MRIs at 1.5 T (18 patients) or 3 T (9 patients) based on a clinical protocol that met or exceeded “Minimum Recommended Imaging” in epilepsy (20) confirmed by both neuroradiologists and epileptologists. Resolution of the clinical scans ranged from 0.85 × 0.85 × 3 mm^3^ at 3 T to 1.2 × 1.2 × 3 mm^3^ at 1.5 T, with variation within each set. The sSOZ was determined independently by two epileptologists (LM, MF) based on EEG and clinical data and all determinations were concordant. Demographic and clinical data is reported is Table 1.

**Table 1:** Patient characteristics.

**Patient number**	**Gender**	**Age at scan**	**Age at diagnosis**	**Laterality**	**Epilepsy type**	**Medication type**
1	Female	33	33	R	mTLE	LEV
2	Male	20	20	R	mTLE	OXC
3	Female	22	20	R	mTLE+	LTG
4	Female	21	18	L	mTLE+	LTG, TPX
5	Female	22	19	L	mTLE	LTG, TPX
6	Male	28	21	L	mTLE	OXC
7	Male	25	15	L	mTLE	LTG, LEV
8	Female	45	35	L	mTLE	ZNS, ESL, CLB
9	Female	33	17	L	mTLE	LEV
10	Female	28	10	R	mTLE+	LTG, OXC
11	Male	29	8	L	mTLE+	CBZ, LEV
12	Female	27	4	L	mTLE	LEV, TPX
13	Male	37	2	L	mTLE	LTG, PHT
14	Male	56	55	L	NE	LTG
15	Female	28	26	L	NE	LEV
16	Male	24	16	R	NE	LCM, LEV
17	Female	28	14	L	NE	LTG, ZNS
18	Male	51	21	R	NE	LCM, OXC, TPX
19	Male	43	43	B/L	mTLE	LEV, OXC
20	Male	33	30	B/L	mTLE	LCM, OXC
21	Female	33	20	B/L	mTLE	CBZ, LEV
22	Male	20	5	B/L	NE	CBZ, LTG, VPA
23	Female	23	17	B/L	NE	LEV, OXC
24	Male	19	13	B/L	NE	OXC, TPX
25	Male	22	13	B/L	NE	VPA, ESL
26	Male	29	28	B/L	NE	LCM
27	Female	56	18	B/L	NE	CBZ, LEV, VPA

*R, right; L, left; B/L, bilateral; mTLE, mesial temporal lobe epilepsy (+, ipsilateral neocortical involement); NE, neocortical epilepsy; CBZ, carbamazepine; LCM, lacosamide; LTG, lamotrigine; LEV, levetiracetam; OXC, oxcarbazepine; PHT, phenytoin; TPX, topiramate; VPA, valproate; ZNS, zonisamide; ESL, eslicarbazepine acetate; CLB, clobazam*.

### MRI Imaging Protocol

The MRI scans performed for all study participants were completed on a 7T whole body scanner (Magnetom, Siemens Healthcare, Erlangen, Germany) using a SC7CD gradient coil (max slew rate = 200 T/m/s, G_max_ = 70 mT/m), with a single channel transmit and 32-channel receive head coil (Nova Medical, Wilmington, MA, USA). The imaging protocol was optimized for detection of structural abnormalities and has already been described in our previous work (21). Briefly, the protocol included a T1-weighted MP2RAGE sequence: TR = 6,000 ms, TE = 5.1 ms, field of view (FOV) = 225 × 183 mm, voxel size = 0.80 × 0.80 × 0.80 mm, *R* = 3, acquisition time = 7:26 min. A T2-weighted turbo spin echo (T2-TSE) sequence was also acquired: TR = 6 000 ms, TE = 69 ms, FOV = 225 × 183 mm, voxel size = 0.4 × 0.4 × 2.0 mm, *R* = 2, acquisition time: 6:14 min. Both were acquired at a coronal-oblique angle, perpendicular to the angle of the long axis of the body of the hippocampus with parallel imaging for acceleration (*R*).

### Qualitative Visual Assessment

Visual assessment of all 7T images were completed by two expert neuroradiologists (BD, PP). For this study, both neuroradiologists were initially blinded to participant status and clinical data, and assessed the 7T images for structural abnormalities. Disagreements were resolved by consensus. Both reviewers were then un-blinded to the sSOZ defined by our study's two expert epileptologists (LM, MF). Correlation between the structural abnormalities found at 7T and the patient's sSOZ was graded on a five-point scale: *none* (no 7T lesions/lesion has no epileptogenic significance); *uncertain* (7T lesion has epileptogenic potential but does not localize to the sSOZ); *possible* (7T lesion of epileptogenic potential which localizes to the patient's sSOZ but is not the definitive cause); *likely* (7T lesion of epileptogenic potential which highly localizes to the sSOZ and is highly likely to cause the patient's epilepsy); *definite* (7T lesion of epileptogenic potential which highly localizes to the sSOZ and is confirmed through surgical intervention) (21). Both neuroradiologists also reported structural asymmetry of the hippocampus according to hemisphere (right > left or left > right) which was subsequently categorized based on sSOZ (ipsilateral > contralateral or contralateral > ipsilateral).

### Quantitative Assessment by Automated Segmentation

**Figure 1:**
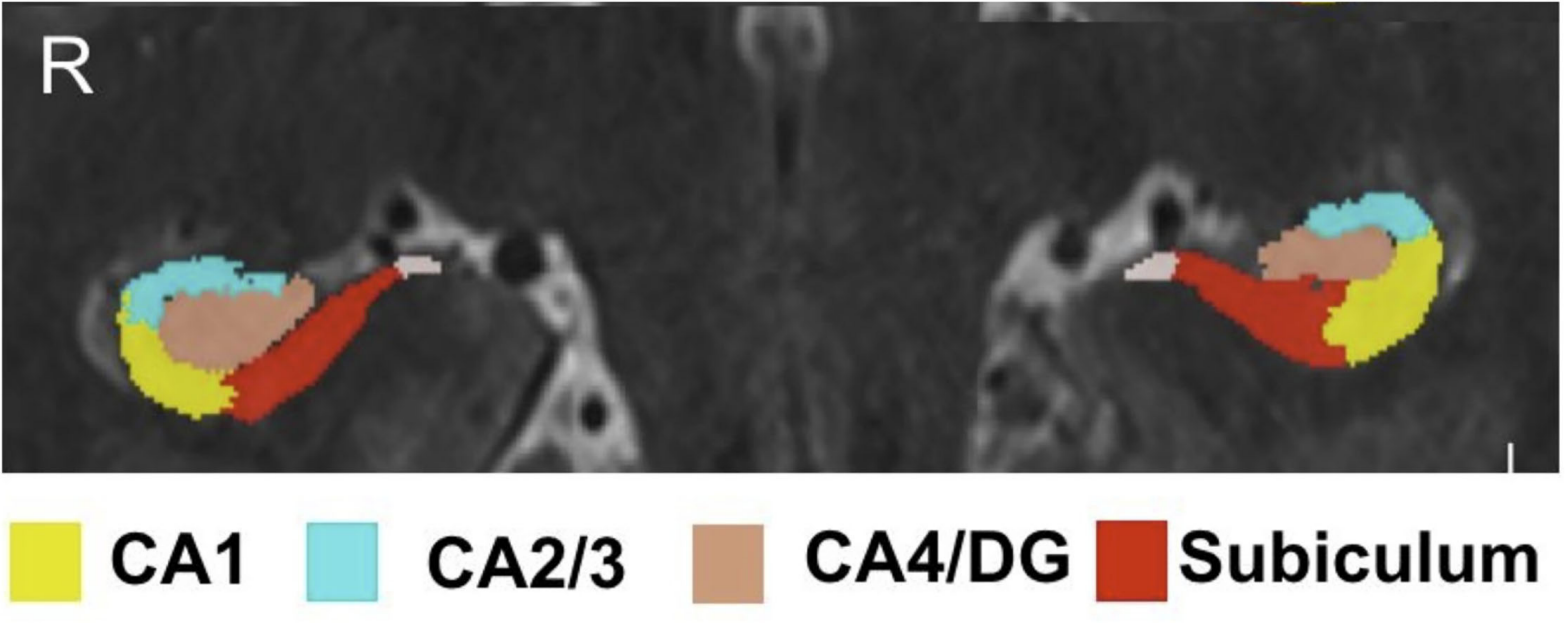
Hippocampal subfield segmentation by ASHS. Subfield segmentation generated by ASHS. The subfields shown include: CA1, CA2/3, CA4/DG, and subiculum.

Hippocampal subfield volumes were acquired by implementing a previously reported software solution (automatic segmentation of hippocampal subfields, ASHS) which uses high resolution T1- and T2-weighted scans as inputs (22). The ASHS algorithm was trained using an atlas of manually traced hippocampi on a separate set of 7T data comprised of 15 subjects without clinically significant, identifiable hippocampal abnormalities. The algorithm was validated through visual inspected by an expert neuroanatomist (PH) and neuroradiologist (BD), the subfields delineated manually and thus by ASHS included CA1, CA2/3, CA4/DG, and subiculum. Automatic segmentation of hippocampal subfields also provided volumetric data on each subfield. Intracranial volume (ICV) was generated using the BrainSeg output provided by FreeSurfer (http://surfer.nmr.mgh.harvard.edu) version 6.0. Both ASHS and FreeSurfer outputs were required through a self-assembling pipeline built in-house. All hippocampal subfield volumes underwent qualitative manual inspection to ensure quality of the segmentation methods (JA). Figure 1 shows an example of subfields on 7T images delineated by ASHS.

### Experimental Design and Statistical Analysis

All patients with unilateral and bilateral mTLE and NE were matched with controls based on age (±4 years) and gender. First, all hippocampal subfields were normalized for head size by total ICV based on the following formula: ${normalized\;volume}_{\left(subject\right)}=\frac{{raw\;volume}_{\left(subject\right)}*1,000,000}{ICV_{\left(subject\right)}}$ (15). In patients with unilateral mTLE and NE, ICV normalized hippocampal subfields were grouped into ipsilateral and contralateral volumes based on the sSOZ (15, 18). For controls and patients with bilateral mTLE and NE, ICV normalized subfields from both hemispheres were averaged for comparison. Regarding normality, a Shapiro Wilk test was performed and the value was >0.05 for all of the subfields. One-way analyses of variance (ANOVA) with group (control, ipsilateral and contralateral mTLE, ipsilateral and contralateral NE, and bilateral onset patients) as independent variables and ICV normalized hippocampal subfields as dependent variables were performed to test for group differences with Bonferroni *post-hoc* tests. Patients with both mTLE and NE were analyzed in the mTLE group.

For patients with unilateral mTLE and NE, asymmetric indices were determined for each ICV normalized hippocampal subfield. For controls, ipsilateral and contralateral subfields were designated based on the sSOZ of their matched patient. Volumetric asymmetry indices were based on the following formula established in the literature $\left(\frac{Contralateral-Ipsilateral}{Contralateral+Ipsilateral}\right)$ (18). In order to assess the severity of asymmetry between different subfields for individual analyses and account for the presence of inter-hemispheric asymmetries in healthy controls, asymmetry indices were converted into z-scores with respect to the corresponding distribution of asymmetries in healthy controls using the following formula: $z-score=\frac{{asymmetric\;index}_{\left(subject\right)}-mean\;{asymmetric\;index}_{\left(controls\right)}}{{standard\;deviation\;of\;asymmetric\;index}_{\left(controls\right)}}$ (15, 18). Consistent with previous studies investigating hippocampal asymmetry (15, 16), an asymmetric index z-score ≥ 1, or measure of asymmetry larger than 1 SD from the mean, was determined to be “considerably asymmetric” and a z-score ≥2, or 2 SDs from the mean, was considered to be severely asymmetric suggesting larger contralateral volumes compared to ipsilateral volumes.

Since longer disease duration has been shown to correlate with greater subfield atrophy, Pearson's two-tailed correlations were completed to determine the strength and direction of association between disease duration and asymmetric index z-scores for patients with unilateral mTLE and NE (17). If any subfields for either the unilateral mTLE or NE cohort were strongly associated with disease duration (*p* < 0.01), separate subgroup analysis was planned to assess the severity of asymmetry between all hippocampal subfields by disease duration. As previously described by Galego et al., for methods in preliminary studies assessing volume change as a function of disease duration, patients would be dichotomized based on the median disease duration for the subgroup (short vs. long) and compared with their respective age and gender-matched controls by multivariate ANOVA (MANOVA) with group as the independent variable (control, short disease duration, long disease duration) and AI z-scores across all hippocampal subfields as the dependent variable. All statistical analyses and relevant figures were completed with MATLAB2018a (Mathworks Inc., Natick, MA, USA) and SPSS Statistics (IBM Corp., Armonk, NY, USA).

## Results

### Automated Segmentation Group Comparisons of Volume

The group comparison analysis suggested there was a statistically significant difference between groups (controls vs. ipsilateral, contralateral and bilateral mTLE and NE) and hippocampal subfield volumes as determined by one-way ANOVA (Table 2). Bonferroni *post-hoc* tests revealed that the ICV normalized CA1 hippocampal subfield volume was statistically significantly smaller only in mTLE patients on both the ipsilateral and contralateral sides compared to controls. The ICV normalized CA2/3 hippocampal subfield volume was statistically significantly smaller only on the ipsilateral side for unilateral mTLE patients compared to controls. The ICV normalized CA4/DG and Subiculum was not statistically smaller in any of the groups. There was no statistically significant difference between controls vs. NE groups and bilateral group across any hippocampal subfield compared to controls.

**Table 2:** Mean (SD) of hippocampal subfield volumes.

	**Controls**	**Unilateral mTLE**		**Unilateral-NE**		**Bilateral mTLE/NE**
	***n* = 27**	***n*** **= 13**		***n*** **= 5**		***n* = 9**
	**Mean**	**Ipsi**	**Contra**	**Ipsi**	**Contra**	**Mean**
CA1	760.3 (86.2)	602.8 (163.9)^*^	632.2 (59.1)^*^	734.0 (50.1)	718.1 (69.3)	658.2 (107.4)
CA2/3	267.2 (51.7)	200.7 (56.1)^*^	242.5 (39.1)	236.5 (35.6)	228.4 (38.9)	231.2 (44.4)
CA4/DG	364.8 (73.7)	322.5 (77.7)	343.7 (57.7)	342.7 (79.6)	316.4 (77.8)	333.9 (85.4)
Sub	815.2 (101.4)	683.8 (148.6)	703.6 (74.2)	757.0 (82.8)	752.5 (66.0)	699.8 (83.4)

*Mean units of subfields with standard deviation (SD)*.

* *p < 0.05 compared to controls. mTLE, mesial temporal lobe epilepsy; NE, neocortical epilepsy; DG, dentate gyrus; Sub, subiculum; Ipsi, ipsilateral; contra, contralateral to the suspected seizure onset zone determined by clinical data and EEG. mTLE*

### Visual Assessment and Automated Segmentation Comparisons for Asymmetry

The qualitative assessment of images at 7T for this population has already been previously presented (21). Briefly, while all 27 patients in this study had non-lesional diagnostic 1.5 or 3 T MRI scans, visual assessment at 7T by two expert radiologists (BD, PP) revealed structural abnormalities not otherwise seen at 1.5 or 3 T in 19 patients (70.4%). Neuroradiologists reported structural asymmetry of the hippocampal volume (R > L or L > R) which was subsequently categorized based on sSOZ (ipsilateral > contralateral or contralateral > ipsilateral). On visual assessment, 9 of 27 patients were determined to have hippocampal asymmetry at 7T.

In order to both compare results from visual assessments and automated segmentation findings and identify which hippocampal subfields are most affected in epilepsy, asymmetric indexes, and corresponding z-scores were determined for patients with unilateral mTLE and NE. Z-scores of ≥1 were hippocampal subfields that were determined to be considerably asymmetric and z-scores ≥2 were considered to be severely asymmetric suggesting larger contralateral volumes compared to ipsilateral volumes (Santyr) (Table 3). Amongst the 13 patents with unilateral mTLE, four patients were determined to have hippocampal asymmetry with the contralateral side greater than the ipsilateral side on visual assessment. All four patients had at least one subfield with either considerable or severe asymmetry in the same direction based on automated segmentation (#12 and #13: severely asymmetric in all subfields, #10: severely asymmetric Subiculum and CA1, #9: considerably asymmetric CA2/3). Automated segmentation detected three additional patients with considerably or severely asymmetric subfields. Two of these three patients only had asymmetry in the CA2/3 region (#5 and #8) with no asymmetry noted on visual assessment.

**Table 3:** Normalized asymmetry index Z-scores.

**Patient number**	**Group**	**Correlation between sSOZ and 7T MRI visual assessment**	**Hippocampal asymmetry on 7T MRI visual assessment**	**Disease duration**	**Hippocampal subfield**			
					**Sub**	**CA1**	**CA2/3**	**CA4/DG**
1	Unilateral mTLE	Uncertain	Ipsi > Contra	0	−0.32	−2.89	−0.53	−1.29
2	Unilateral mTLE	Uncertain	Ipsi > Contra	0	0.18	−1.28	−0.60	−1.18
3	Unilateral mTLE	Uncertain	Ipsi > Contra	2	2.15^**^	−2.13	−1.19	−1.08
4	Unilateral mTLE	None	–	3	−0.45	−0.53	0.58	0.05
5	Unilateral mTLE	None	–	3	0.62	−0.27	1.11^*^	0.55
6	Unilateral mTLE	None	–	7	−1.33	−1.62	0.62	−1.21
7	Unilateral mTLE	Uncertain	–	10	−0.90	−1.74	0.08	−0.46
8	Unilateral mTLE	Uncertain	–	10	−0.11	0.58	2.03^**^	0.23
9	Unilateral mTLE	Likely	Contra > Ipsi	16	−1.15	−0.71	1.18^*^	−0.94
10	Unilateral mTLE	Possible	Contra > Ipsi	18	3.63^**^	4.83^**^	0.16	0.48
11	Unilateral mTLE	Possible	–	21	0.59	−1.73	−0.54	0.00
12	Unilateral mTLE	Likely	Contra > Ipsi	23	6.44^**^	8.53^**^	3.75^**^	3.98^**^
13	Unilateral mTLE	Possible	Contra > Ipsi	35	3.14^**^	8.31^**^	3.78^**^	4.08^**^
14	Unilateral NE	None	–	1	−0.26	−1.07	−0.05	−1.50
15	Unilateral NE	Uncertain	Contra > Ipsi	2	−0.10	0.14	0.64	1.06^*^
16	Unilateral NE	Definite	–	8	1.69^*^	−1.25	−0.64	−0.05
17	Unilateral NE	None	–	14	−0.41	0.21	−0.42	−1.72
18	Unilateral NE	Possible	Ipsi > Contra	30	1.41^*^	−1.15	−0.88	−2.09

*Correlation between sSOZ (defined by EEG and clinical data) and 7T MRI visual assessment was defined by five categories from none to uncertain, possible, likely, and definite localization of 7T lesion to sSOZ. Hippocampal Asymmetry was documented by hemisphere (R > or < L) but subsequently transformed to ipsilateral or contralateral based on sSOZ*.

* *Subfields with z-scores >1 (considerably asymmetric)*.

** *Subfields with z-scores >2 (severely asymmetric)*.

*mTLE, mesial temporal lobe epilepsy; NE, neocortical epilepsy; DG, dentate gyrus; Sub, subiculum; sSOZ, suspected seizure onset zone; Ipsi, ipsilateral; Contra, contralateral*.

Interestingly, four patients (#3, #9, #16, and #18) had subfields that were considerably or severely asymmetric in opposite directions. For example, patient #3 was noted to have overall hippocampal asymmetry on visual assessment, reporting that the contralateral side was in fact smaller than the ipsilateral. This was further explained by the automated segmentation results at the subfield level as patient #3 was found to have a severely asymmetric subiculum, smaller on the ipsilateral side (z-score of >2), but all other subfields were asymmetric, but smaller on the contralateral side (z-scores of < -1 or −2).

Amongst the five patients with unilateral NE, only one patient was determined to have hippocampal asymmetry of contralateral greater than ipsilateral on visual assessment (#15). This patient had considerable asymmetry in the CA4/DG region (z-score 1.06). Two additional patients with asymmetric subfields were detected by automated segmentation. Both had considerable asymmetry in the subiculum, one un-detected on visual assessment (#16). The second patient (#18) was reported to have whole hippocampal asymmetry on visual assessment, with the contralateral side actually smaller than ipsilateral. However, this was similar to patient #3, as patient #16 had considerable asymmetry in the subiculum, smaller on the ipsilateral side (z-score of >1), but other subfields (CA1 and CA4/DG) were asymmetric, but smaller on the contralateral side (z-scores of < -1 or −2).

### Correlation Between sSOZ and 7T MRI Visual Assessment by Disease Duration

While the correlation of new findings detected at 7T to the sSOZ has been previously described, this study also assessed the relationship of ratings *none, uncertain, likely, possible*, and *definite* based on disease duration (Table 3). In the 13 patients with unilateral mTLE, when neuroradiologists were unblinded to the sSOZ as determined by epileptologists from clinical and EEG data, the correlation of structural abnormalities identified at 7T and the sSOZ was rated as *none* or *uncertain* in eight patients of which all patients had a disease duration of 10 years or less. In all five unilateral mTLE patients rated as *likely* or *possible*, patients had a disease duration of >10 years. No patients were rated as definite since none had confirmed surgical intervention at the time of analysis. In the five patients with unilateral NE, correlation ratings between structural abnormalities identified at 7T and sSOZ did not follow similar patterns with disease duration.

### Correlation of Asymmetric Index and Disease Duration

**Figure 2:**
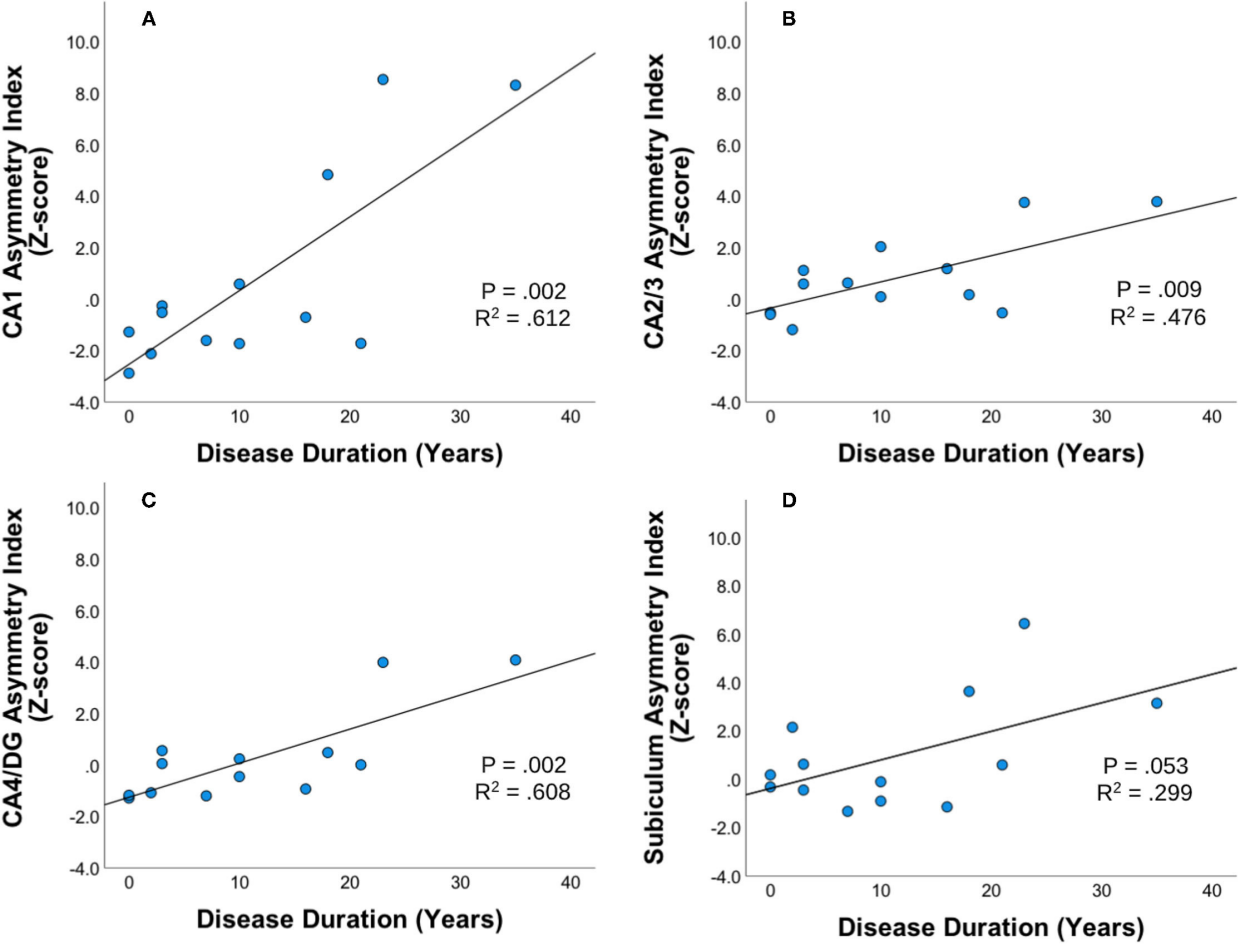
Disease duration and subfield asymmetry. Strength and direction of association between disease duration and asymmetric index Z-scores across hippocampal subfields **(A)** CA1, **(B)** CA23, **(C)** CA4DG, and **(D)** Subiculum for patients with unilateral mTLE (mesial temporal lobe epilepsy). Pearson's two-tailed correlations were completed. Correlation was significant if *p* < 0.01.

Statistically significant correlations were found between asymmetric index z-scores across hippocampal subfields within the unilateral mTLE group using Pearson two-tailed correlations (*p* < 0.01). In the unilateral mTLE group, the CA1, CA2/3, CA4/DG hippocampal subfields and disease duration had a statistically significant positive relationship such that the asymmetric index z-score increased as patients had a longer disease duration (Figure 2). While the subiculum in unilateral mTLE followed a similar trend, it did not reach significance (*p* = 0.053). No statistically significant correlations between disease duration and hippocampal subfield asymmetry were found within the NE group.

### Asymmetric Index Comparisons by Disease Duration in mTLE

**Figure 3:**
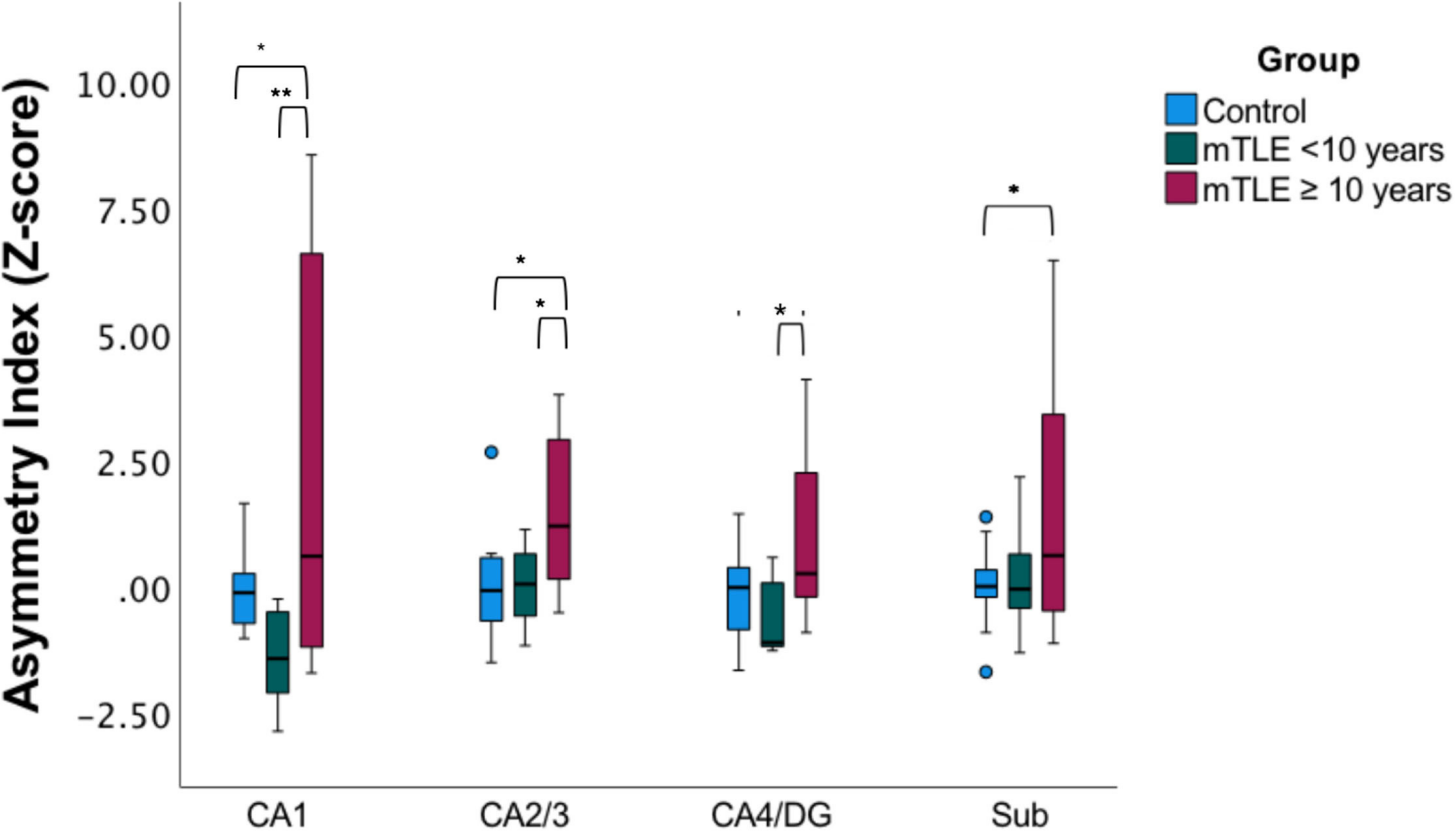
Subfield asymmetry by duration cohorts. Asymmetric index Z-scores across hippocampal subfields in age-matched and gender-matched controls, mTLE cases with a disease duration of <10 years, (median of the mTLE cohort), and mTLE cases with a disease duration ≥ 10 years. *p < 0.05, **p < 0.01.

Since only unilateral mTLE patients exhibited significant correlation with disease duration a subgroup analysis was performed in this cohort based on the median disease duration for the subgroup (Figure 3) (23). There was a statistically significant difference in asymmetry index z-scores between groups (control, mTLE disease duration <10 years, and mTLE disease duration ≥10 years) as determined by one-way MANOVA [*F*_(8,40)_ = 2.503, *p* = 0.026, Wilk's Λ = 0.444]. No significant differences in asymmetric index z-scores were seen between the control group and the mTLE cohort with a shorter disease duration (<10 years) across all hippocampal subfields. However, when the controls were compared to mTLE patients with longer disease duration (≥10 years), significant differences were observed in the CA1, CA2/3, and subiculum region. Asymmetry was also significantly different within patients cohorts, as the CA1, CA2/3, and CA4/DG regions were significantly different between the shorter and longer disease duration groups.

## Discussion

### 7T Improves Visualization of Structural Abnormalities

In patients with refractory focal epilepsy, accurate localization of seizure foci remains challenging as 20–30% of patients remain MRI-negative at conventional field strengths (24). Our results suggests that in unilateral mTLE patients, the increased SNR and contrast available at 7T (19) may provide novel evidence of structural abnormalities that correlate with clinical and EEG findings (sSOZ) that would be otherwise undetected at 1.5 or 3 T, specifically in mTLE patients with longer disease durations. Since patients with well-defined lesions evident on pre-operative imaging are more likely to have better post-operative outcomes (24), our results suggest 7T may provide valuable information for pre-operative planning in this subset. In patients with a newer diagnosis of mTLE, our data suggests that the marginal benefit of utilizing 7T MRI may be limited.

### Automated Segmentation Improves Detection of Asymmetry in Hippocampal Subfields

While visual analysis at 7T detected overall hippocampal asymmetry not otherwise seen at conventional field strengths, the application of our segmentation technique highlights how delineation of subfields may provide additional information not seen on visual assessment. Subfield level data on structural asymmetry may be particularly important in pre-surgical planning since different patterns of neuronal loss seen in histopathologic studies of hippocampal subfields have been shown to be predictive of post-surgical outcome (3). Additionally, while previous reports of volumetric segmentation at 7T have utilized manual segmentation methods (16, 17), our application of automated segmentation highlights the possibility for reproducibility in studies with larger cohorts. While preliminary manual inspection and segmentation by an experienced-rater increases validity, implementation of automated segmentation can reduce both inter-rater and intra-rater variability, and costs associated with time-burden (25).

### Burden of Disease Duration Affects Hippocampal Subfield Asymmetry in mTLE Patients

Prior work has suggested that disease duration in MRI-positive mTLE patients can cause progressive hippocampal atrophy (3, 12, 26). In studies that included pathology, hippocampal neuronal cell loss was found to underlie the diminished volumes (27, 28). Similarly in our study, disease duration was found to have a statistically significant positive relationship with hippocampal asymmetry within the unilateral mTLE cohort. Importantly, this is the first analysis of subfield-level hippocampal asymmetry at 7T and disease duration in mTLE patients with clinically normal MRIs. Our subgroup analysis comparing controls and shorter vs. longer disease duration based on the median disease duration for the subgroup revealed significant differences in asymmetry in the CA1, CA2/3, and subiculum when controls were compared to patients with longer disease durations. It is possible that on the continuum of patients with mTLE, MRI-negative patients follow similar patterns as MRI-positive patients but with more subtle structural changes that are otherwise un-detected at conventional field strengths. Increased hippocampal atrophy with disease duration was not found in the patients with NE, suggesting that if the hippocampus is not the SOZ, it is not as vulnerable to damage with ongoing seizures.

### Hippocampal Volume Loss and Structural Asymmetry Exists in MRI-Negative Unilateral mTLE

From our application of ultra-high field MRI with automated segmentation, we observed group differences in ICV normalized volume of the CA1 and CA2/3 regions. In patients with unilateral mTLE, significant CA1 volume loss was demonstrated ipsilateral and contralateral to the sSOZ and CA2/3 volume loss only ipsilateral to the sSOZ compared to controls. While this cohort is heterogenous, our subject level quantitative analysis of structural asymmetry validates these results particularly for those with a longer disease duration and is in agreement with typical atrophy patterns described by multiple histopathological studies from MRI-positive patients (3, 6, 13). In HS type 1, which represents 60–80% of cases, the CA1 segment is most severely affected but significant neuronal cell loss is also seen across all subfields. Atypical variants predominantly affect only one subfield such as the CA1 in HS type 2 and CA4 in HS type 3 (3). In our subject-level analysis of mTLE patients with otherwise normal clinical MRIs, we identified considerably or severely asymmetric subfields in 7/13 patients. Notably, two (#12 and #13) patients exhibited patterns most similar to HS type 1 as they were severely asymmetric across all subfields, with the CA1 most severely affected. Two patients with mTLE (#3 and #8) had some subfields that were smaller and some that were larger ipsilateral to the sSOZ. This level of detail has not previously been available and further study is needed.

To date, only two studies have reported comparisons of subfield volumes at 7T in mTLE patients with normal clinical MRIs, however, both utilized manual segmentations and report discordant outcomes. In a subject-level analysis by Santyr et al. (16), 1/9 patients exhibited severe ipsilateral volume loss in the CA4/DG and 2/9 exhibited considerable atrophy of the CA2/3 contralateral to the sSOZ. However, subfields were segmented within the anterior body of the hippocampus which may have underestimated atrophic changes in the head and posterior body. Subsequently, Voets et al. (17), who extended subfield measurements to the full hippocampus, demonstrated subfield atrophy in the ipsilateral CA3 region in 4/7 patients. An additional report on hippocampal asymmetry at 7T for MRI-negative mTLE patients revealed only whole hippocampal asymmetry without reaching significance at the subfield level (18). Due to the heterogeneity in volumetric loss and asymmetry patterns, these preliminary studies have suggested conventional strength MRI-negative mTLE patients may represent a separate pathophysiological process. While our results are also heterogenous, we show some patients, especially those with longer disease durations, may follow similar atrophy or asymmetry patterns typical of MRI-positive patients. Ultimately, our group-level comparisons should be interpreted with caution given the variability observed at the individual-level analysis. Future work should focus on increasing cohort sizes with similar characteristics, such as disease duration, in order to validate these results.

### Atrophy Patterns in Neocortical Epilepsy and Bilateral Onsets Remain Difficult to Characterize

Our preliminary analysis included patients with unilateral and bilateral mTLE and NE. We observed group differences in hippocampal subfield volumes for the CA1 region and CA2/3, however, *post-hoc* analyses revealed no significant difference between patients with unilateral NE and controls. While the majority of patients with epilepsy have seizures originating in the mesial structures of the temporal lobe, primarily within the hippocampus, approximately 30% patients have seizures arising from the neocortical regions outside the hippocampus (29). Although there is a strong interconnection between the neocortex and hippocampus, previous studies have had contradictory results regarding the effect of repeated seizures originating in the neocortex and resultant metabolic abnormalities due to secondary damage to the hippocampus (29, 30). Our results from a small cohort of patients with unilateral NE do not provide evidence to suggest secondary atrophy of the hippocampus.

Similarly, *post-hoc* analyses in group differences in hippocampal subfields did not reveal statistically significant volume reduction in patients with bilateral onsets (mTLE/NE) and controls. However, our methods included the averaging of subfield volumes across both sides and assumed there were no side effects between groups. This method in part was chosen since no pronounced asymmetry was seen on visual analysis of 7T images, however, future studies should include investigation of asymmetry at the subject-level.

### Study Limitations

While we report the application of ultra-high field 7T MRI and automated segmentation in the largest cohort of epilepsy patients to date, the low number of participants for relevant subgroups is a limitation to this study. This is largely secondary to the logistical challenge of scanning patients already non-lesional at 1.5 or 3 T. Although we have identified similar patterns between MRI-negative and MRI-positive patients, our cohort demonstrates the heterogeneity in cases and subfield patterns. Future studies will include larger cohorts such that greater statistical power can be achieved in assessing various types of epilepsy including mTLE and NE. Secondly, the determination of lateralization of sSOZ and structural asymmetry was determined based on consensus agreement between epileptologists interpreting clinical and EEG data. Validation of the sSOZ with post-surgical histopathological studies will assist in confirming seizure foci. Histopathological studies will help provide critical information whether MRI-negative epilepsy is indeed a distinct histological entity. Future investigations will also include post-surgical clinical outcomes. While we have demonstrated the application of 7T and segmentation methods to identify affected subfields, its utility assisting pre-operative planning and improving clinical outcomes remains unknown. Another limitation of this study is that the clinical MRI at lower field strength (either 1.5 or 3 T) was obtained at various institutions with varying protocols and quality. For that reason, it is not possible to determine if the segmentation and thus these results is only possible with the higher resolution 7T MRI. Future study designs should consider a 3 and 7T MRI with similar acquisition protocols for a more direct comparison.

## Conclusions

Although patterns in hippocampal subfield atrophy patterns have been widely investigated in patients with evidence of HS on conventional strength MRIs, few studies have demonstrated the application of ultra-high field 7T MRI in epilepsy patients that are otherwise considered MRI-negative at 1.5 or 3 T. A custom imaging protocol was developed to produce high resolution T2-TSE and T1-weighted MP2RAGE images. Images were qualitatively assessed and results were quantitatively analyzed based on volumes and asymmetry index z-scores from an automated segmentation algorithm that was trained, validated, and then generated through a self-assembling pipeline built in house. This study highlights the ability of 7T to provide novel qualitative information including sSOZ lateralization and value of combined and novel findings with the application of automated segmentation in volumetric atrophy and structural asymmetry in hippocampal subfields CA1, CA2/3, CA4/DG, and the subiculum. Additionally, we report the first application of 7T with automated segmentation to characterize the relationship between disease duration burden and asymmetry across hippocampal subfields in mTLE patients who are MRI-negative at conventional field strengths. Further investigation of MRI-negative epilepsy at ultra-high field 7T MRI is warranted.

## Data Availability Statement

The raw data supporting the conclusions of this article will be made available by the authors, without undue reservation.

## Ethics Statement

The studies involving human participants were reviewed and approved by Institutional Review Board at Mount Sinai Hospital. The patients/participants provided their written informed consent to participate in this study.

## Author Contributions

AP: design and conceptualized study, interpretation of data, and drafted the manuscript for intellectual content. LM: design and conceptualized study, acquisition and interpretation of data, and drafted the manuscript for intellectual content. BD: major role in the acquisition of data. JA, JR, RF, PH, MF, and JY: major role in the acquisition of data and interpretation of data and revised the manuscript for intellectual content. PB: design and conceptualized study and revised the manuscript for intellectual content.

## Funding

This work was supported by the National Institute of Health—National Institute of Neurological Disorders and Stroke (R00 NS070821) and National Institute of Health—National Institute of Mental Health (R01 MH109544).

## Conflict of Interest

PB (the principal investigator in this study) is a named inventor on patents relating to magnetic resonance imaging (MRI) and RF pulse design. The patents have been licensed to GE Healthcare, Siemens AG, and Philips international. PB receives royalty payments relating to these patents. PB is a named inventor on patents relating to slice-selective adiabatic magnetization T2-preparation (SAMPA) for efficient T2-weighted imaging at ultrahigh field strengths, methods for producing a semi-adiabatic spectral-spatial spectroscopic imaging sequence and devices thereof, and semi-adiabatic spectral-spatial spectroscopic imaging. RF is a named inventor on patents relating to magnetic resonance imaging (MRI) and RF pulse design, semi-adiabatic spectral spatial spectrospatial imaging (SASSI) and semi-adiabatic matched phase spin echo power independent of the number of pulses (SEAMS PINS). She does not receive financial compensation related to these patents. The remaining authors declare that the research was conducted in the absence of any commercial or financial relationships that could be construed as a potential conflict of interest.

## Publisher's Note

All claims expressed in this article are solely those of the authors and do not necessarily represent those of their affiliated organizations, or those of the publisher, the editors and the reviewers. Any product that may be evaluated in this article, or claim that may be made by its manufacturer, is not guaranteed or endorsed by the publisher.
